# Anthropogenic Litter in Urban Freshwater Ecosystems: Distribution and Microbial Interactions

**DOI:** 10.1371/journal.pone.0098485

**Published:** 2014-06-23

**Authors:** Timothy Hoellein, Miguel Rojas, Adam Pink, Joseph Gasior, John Kelly

**Affiliations:** Department of Biology, Loyola University Chicago, Chicago, Illinois, United States of America; Uppsala University, Sweden

## Abstract

Accumulation of anthropogenic litter (i.e. garbage; AL) and its ecosystem effects in marine environments are well documented. Rivers receive AL from terrestrial habitats and represent a major source of AL to marine environments, but AL is rarely studied within freshwater ecosystems. Our objectives were to 1) quantify AL density in urban freshwaters, 2) compare AL abundance among freshwater, terrestrial, and marine ecosystems, and 3) characterize the activity and composition of AL biofilms in freshwater habitats. We quantified AL from the Chicago River and Chicago's Lake Michigan shoreline, and found that AL abundance in Chicago freshwater ecosystems was comparable to previously reported data for marine and terrestrial ecosystems, although AL density and composition differed among habitats. To assess microbial interactions with AL, we incubated AL and natural substrates in 3 freshwater ecosystems, quantified biofilm metabolism as gross primary production (GPP) and community respiration (CR), and characterized biofilm bacterial community composition via high-throughput sequencing of 16S rRNA genes. The main driver of biofilm community composition was incubation location (e.g., river vs pond), but there were some significant differences in biofilm composition and metabolism among substrates. For example, biofilms on organic substrates (cardboard and leaves) had lower GPP than hard substrates (glass, plastic, aluminum and tiles). In addition, bacterial communities on organic substrates were distinct in composition from those on hard substrates, with higher relative abundances of bacteria associated with cellulose decomposition. Finally, we used our results to develop a conceptual diagram designed to unite the study of AL in terrestrial and freshwater environments with the well-established field of marine debris research. We suggest this broad perspective will be useful for future studies which synthesize AL sources, ecosystem effects, and fate across multiple ecosystem types, and will benefit management and reduction of global AL accumulations.

## Introduction

Accumulation of anthropogenic litter (i.e. garbage; AL) in marine environments has received increased attention in the popular press and scientific literature in recent years [1]–[3]. Researchers have documented large AL accumulations in pelagic gyres of the Pacific and Atlantic Oceans, which have earned such titles as “Pacific trash vortex” and “plastic soup” [3], [4]. Images of AL accumulations on beaches, and ingestion of plastic and styrofoam by fish, marine birds and turtles have also increased public awareness [5]–[7]. Finally, the release of toxic compounds and microscopic particles via plastic decomposition in the environment has garnered attention [8], [9].

Current research on AL in the ocean (i.e. marine debris) identifies two primary sources: direct inputs from boats and anglers, and inputs from terrestrial and riverine ecosystems [10]. However, the patterns of AL abundance, retention, transformation and transport within freshwater ecosystems are unknown, and are likely a significant component of the global AL ‘life cycle’. For example, because rivers can transport AL between terrestrial and marine environments, AL retention or transformation in rivers can attenuate its delivery to downstream ecosystems. From a management perspective, freshwaters may serve as easier collection sites for AL relative to marine coastal and pelagic zones. Therefore, empirical analyses of AL pools, fluxes, and interactions with biota in freshwaters are needed to understand its ecosystem effects and to develop management strategies for the global concern of AL accumulation [11].

When AL enters aquatic habitats it will become rapidly colonized by microbial biofilms composed of bacteria, fungi, and algae in an extracellular mucilaginous matrix. Biofilms are complex, taxonomically diverse communities which support a wide range of metabolic activities [12]. Biofilms can play important roles in nutrient cycling within aquatic habitats [13], and they can also serve as an important food source for higher trophic levels, such as invertebrates and fish [14]. Therefore, biofilm colonization often serves as the initial biotic interaction of solid substrates in aquatic environments, and this interaction can be ecologically significant.

Substrate composition can have a strong effect on biofilm community composition and activity [15], [16]. In addition, differences in substrate quality (e.g., nitrogen content) and mobility drive rates of nutrient demand, metabolism, and community structure within biofilms [16], [17]. Recent evidence indicates microplastics in the open ocean support microbial biofilms that are distinct in composition from the microbial community of surrounding waters [18]. The pattern suggests that the plastic surfaces selected for a unique microbial community, which may carry out different metabolic pathways than those in the open water, including plastic decomposition. However, patterns of biological activity and community composition of biofilms colonizing AL have not previously been measured in freshwaters.

Our objectives for this study were to 1) measure AL abundance in urban freshwaters, 2) compare AL abundance across terrestrial, freshwater, and marine ecosystems, and 3) measure biofilm activity and bacterial community composition on natural and AL substrates in freshwater ecosystems.

## Methods

### Anthropogenic litter collection

We sampled three 70–100-m reaches in the North Branch of the Chicago River and adjacent riparian zone, on 3 different days in summer 2011. Study reaches were in the Bunker Hill Forest Preserve in Chicago, IL (latitude 41.999273, longitude −87.779868), and were spaced approximately 250-m apart. The North Branch of the Chicago River watershed area is 110 km^2^, and the land-use is largely urban and suburban with very little agriculture [19]. In each reach, we removed all AL from the river benthos (width = 18–23 m), and all AL from one bank on the riparian zone within 10-m of the water's edge. We also collected AL from three 400-m reaches of Lake Michigan beaches adjacent to the lakeshore campus of Loyola University Chicago (LUC), located in the Rogers Park neighborhood of Chicago, IL (latitude 42.002541, longitude −87.656464). We sampled the beaches on 3 separate days in summer 2011 by establishing a 400-m transect parallel to the shore and collecting all AL within 50-m from the water line. AL <1 cm was not collected, so this analysis does not include microplastic particles. In the laboratory, we removed dirt from the AL, sorted it by category, and let it air dry for several days. We weighed and measured surface area of each item from its length and mean width, and calculated AL density as the number, mass, and surface area of AL per area searched (No. m^−2^, g m^−2^, and AL cm^2^ m^−2^). Field studies did not involve endangered or protected species at any of the locations of our study. The Forest Preserve District of Cook County issued permission for research at the North Branch of the Chicago River. No permission was required for litter collection at the Lake Michigan beach. The Land Management Committee issued permission for research at the pond on private land at the Loyola University Retreat and Ecology Campus (LUREC; see below). Future permissions for work at that site can contact the campus office at (815) 338–1032.

### Anthropogenic litter density in freshwater vs marine ecosystems

We compared our results to AL abundance from published studies conducted in marine beaches. Comparisons among AL studies are complicated by variability in the units used to quantify AL abundance and the categories used to report AL types. AL density in the literature is reported as relative proportion (%), the number of items collected along a transect (No. m^−1^), or the number of items per area searched (No. m^−2^). AL density by mass (i.e, g m^−2^ or g m^−1^) is infrequently reported [1], [20]. Studies often classify AL types according to different schemes, which are either based upon material composition (e.g., metal, plastic, and glass), identity (e.g., cigarettes, plastic bags, tires), or function (e.g., fishing, food-related, and construction) [1], [21], [22]. To facilitate comparison among our data and literature values, we first compared AL abundance and mass (No. m^−1^ and g m^−1^) of individual AL categories between our sites and studies from marine beaches which used similar units, categories, and did not include microplastic abundance. These included sites in Brazil [23], New Jersey USA [24], South Africa [25], and Oman [26]. The majority of AL studies report abundance as the relative proportion of different categories present. We compared relative amounts of AL categories across habitats including city blocks [27], marine beach [28], lake beach (this study), riparian zone (this study), shallow marine benthos [1], river benthos (this study), and marine offshore benthos [29]. The selected studies reflect the global nature of AL research, however, we note the selected sites are examples of each habitat, and do not represent mean AL composition of the different ecosystems.

### Litter incubation and biofilm activity

To measure biofilm activity and community composition on AL and natural surfaces, we selected 4 of the most common types of AL (i.e. glass, aluminum, plastic and cardboard) and used leaves and ceramic tiles (a common surrogate for rocks) to represent natural substrates. We categorized leaf and cardboard as organic substrates and glass, aluminum, tile and plastic (a synthetic organic material) as hard substrates. Polypropylene mesh bags (38×20 cm) with 5 mm openings (Cady Bag Company, Pearson GA) were used to incubate AL and natural substrates in the North Branch of the Chicago River, a 1.4 hectare pond at LUREC in Woodstock, IL (latitude 42.288834, longitude −88.366064), and in the artificial stream facility at LUC. Artificial streams were re-circulating chambers with a paddle wheel, where channel width is 14.0 cm and total flowpath length is 2.0 m [30]. Streams were refilled with 60 L of tap water that had been allowed to dechlorinate for a minimum of 2 d prior to adding to streams, and water level was marked and maintained throughout the study. On the outside of each bag we attached 3 unglazed porcelain tiles and 3 glass tiles (tile size = 25 cm^2^). We drilled a 0.3 cm^2^ hole in each tile using a diamond drill bit and attached the tiles to the bags using cable ties. We cut 6×5.5 cm^2^ pieces from aluminum cans from cola beverages and 20 oz. plastic drinking bottles (polyethylene terephthalate) and attached these to the outside of litterbags with cable ties (N = 3 of each type). Cans and bottles were collected from recycling containers on campus and thoroughly rinsed. Aluminum was situated such that the surface that was previously inside the can was exposed for biofilm growth (i.e. the non-painted surface). Inside the bags, we placed 3 pieces of corrugated, non-waxed or colored cardboard (170 cm^2^) and 3 naturally senesced red maple (*Acer rubrum*) leaves. The cardboard and leaves were separated from each other inside the bag using cable ties. Leaves and cardboard were situated inside the bags, which may have reduced primary production and grazing relative to substrates inside the bag.

Five replicate bags were attached to the benthos of the North Branch Chicago River and LUREC pond in early summer 2012. Bags were incubated from May 7–August 13, 2012 (98 days) in the river and June 1–July 20, 2012 (50 days) in the LUREC pond. In the artificial stream, we placed 25 replicate bags starting on June 8, 2012. We collected 3 replicate bags (1 bag from each artificial stream) on days 7, 27, 34, 48, and 52. When bags were removed from the river and pond, individual substrates were placed in 160 mL specimen containers filled with site water. We collected an additional 6–8 L of unfiltered site water for measurements of GPP and CR. Substrates and site water were kept on ice until returned to the lab, and metabolism measurements were started on the same day, within 2–4 hours of collection. For substrates from artificial streams, we placed substrates directly in specimen containers and began metabolism measurements straightaway.

We measured CR and net primary production (NPP) using the light/dark method [16], [31]. Each specimen container was filled with unfiltered stream water of known dissolved oxygen (DO) concentration and a single substrate (3 replicates of each substrate; N = 18 for the pond and artificial streams, N = 12 for the river). Containers were capped so no air bubbles were present. All containers were placed in an environmental chamber at 22–23°C with constant light. Final DO and incubation time were recorded after 2–4 h in the light. The containers were refilled with fresh site water and incubated in an environmental chamber in the dark for 2–4 h, after which final DO and time were recorded. Three ‘blank’ containers with site water only were incubated for both NPP and CR measurements to account for metabolism of water column organisms or abiotic changes in DO. Running GPP and CR in sequence could affect results due to cumulative bottle effects, but these were minimized by the short duration of each measurement period. All substrates were immediately frozen at −20°C after metabolism measurements were completed. We calculated metabolism as change in DO in the light (NPP) and dark (CR), and gross primary production (GPP) as NPP - CR (units: µg O_2_ cm^−2^ h^−1^). For leaves and cardboard, we measured surface area by tracing each piece onto paper. The pieces were cut-out and weighed. We weighed paper of known surface area, and used simple linear regression to develop a mass to surface area conversion [15].

### Biofilm community composition

Biofilm was scraped from each substrate incubated in the pond, river, and artificial stream using a sterile razor blade and collected in 2 mL microcentrifuge tubes. For the artificial stream biofilms, we collected biofilm only from substrates sampled on the final incubation date. Genomic DNA was isolated from each biofilm sample (N = 18 in the artificial stream and pond, N = 12 in the river) with the PowerBiofilm DNA Isolation Kit (MoBio Laboratories, Inc., Carlsbad, CA USA) according to manufacturer's instructions. Isolated DNA was placed in 100 µL of a 20 mM Tris solution and stored at −20°C. DNA from each sample was sent to Argonne National Lab for massively parallel, paired-end sequencing of partial 16S rRNA genes using the Illumina MiSeq platform. PCR amplification was performed using primers 515F and 806R to amplify the V4 region of the 16S rRNA gene [32]. PCR amplification failed for 2 samples (1 aluminum and 1 glass substrate from the pond) due to low DNA yield from low biomass biofilms, so no sequencing data were obtained for these samples. Sequencing was conducted as described previously [32]. Sequences were processed using MOTHUR v.1.30.1 [33] as described previously [34]. Briefly, any sequences with ambiguities or homopolymers longer than 8 bases were removed from the data set. Sequences were aligned using the SILVA-compatible alignment database available within MOTHUR. Sequences were trimmed to a uniform length of 253 base pairs and chimeric sequences were removed using Uchime [35]. We classified sequences using the MOTHUR-formatted version of the RDP training set (v.9) and any unknown, chloroplast, mitochondrial, archaeal and eukaryotic sequences were removed. Sequences were clustered into operational taxonomic units (OTUs) based on 97% sequence identity. To avoid biases associated with uneven numbers of sequences across samples, the entire dataset was randomly subsampled to 17,000 sequences per sample.

### Data analysis

We used 1-way ANOVA to compare AL abundance across 3 ecosystem types: Chicago River benthic zone, riparian zone, and Lake Michigan beach. For substrates incubated in the Chicago River and LUREC pond, we used a 1-way ANOVA to compare GPP and CR across AL types. 2-way repeated measures ANOVA (RM-ANOVA) was used to compare GPP and CR across substrates and dates during incubation in the artificial streams.

The compositions of the bacterial communities from individual samples were compared by calculating dissimilarities based on the theta index [36] and visualizing the resulting distance matrix using non-metric multidimensional scaling (nMDS) run within MOTHUR. The significance of differences in theta index scores between sites and substrates was assessed by ANOSIM run within MOTHUR. ANOSIM reports R statistics and p values for comparisons of pairs of groups. Briefly, R = 0 when there are no differences between groups, R approaches 1 as groups become more distinct, R = 1 when all samples in different groups are more dissimilar to each other than any samples in the same group, and the p value indicates the statistical significance of the R statistic [37]. Bacterial families making the most significant contributions to differences in composition between sites and substrates were identified by SIMPER analysis run in Primer V.5 (Primer-E Ltd., Plymouth, United Kingdom). We used two-way ANOVA to assess the effects of sites and substrates on the total number of OTUs identified, the relative abundance of the bacterial families identified by SIMPER, and on the most abundant bacterial phyla. MOTHUR was also used to calculate Inverse Simpson and Shannon (H′) diversity indices [38]. We used one-way ANOVA to compare diversity among substrates in each habitat type. All ANOVAs were completed in SYSTAT 13.0 (Systat, Inc. Chicago, IL), with significant ANOVA (p<0.05) followed by Tukey's multiple comparison tests.

### Data Sharing

All of the sequence data analyzed in this paper can be downloaded from the National Center for Biotechnology Information (NCBI) Sequence Read Archive (SRA) with accession number SRP042298.

## Results

### Anthropogenic litter abundance across freshwater ecosystem types

**Figure 1:**
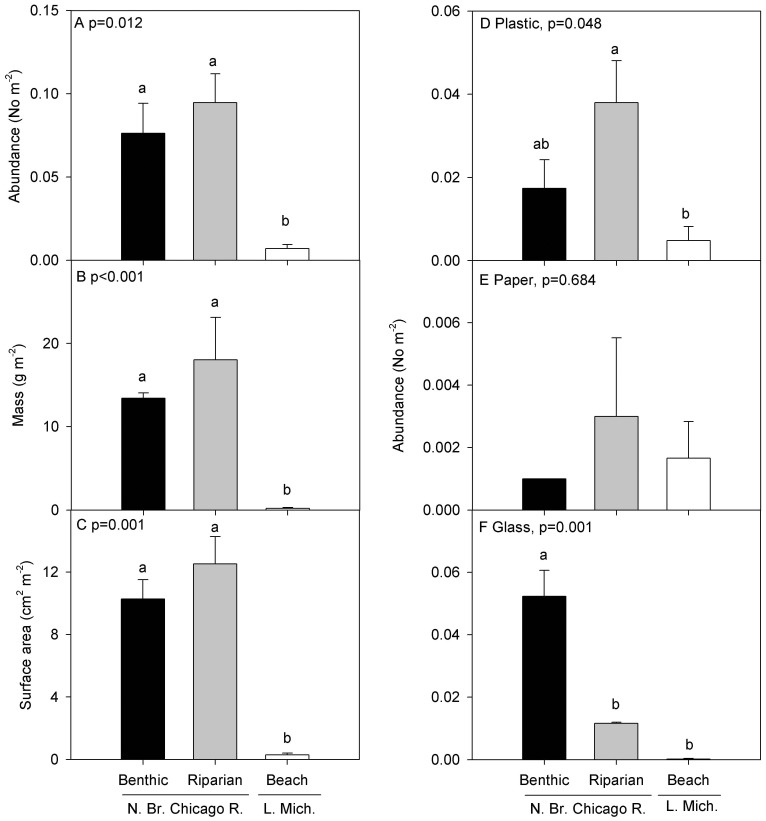
Mean (±SE) amounts of total anthropogenic litter (AL) in the benthic and riparian zones of the North Branch of the Chicago River and a Lake Michigan beach in Chicago, IL, USA by (A) abundance (number m^−2^), (B) mass (g m^−2^), (C) surface area (cm^2^ m^−2^), and by individual categories including abundance of (D) plastic, (E) paper, and (F) glass.

Lake Michigan beaches had significantly less AL relative to the Chicago River riparian and benthic zones when AL abundance was expressed as number of items, mass, and surface area (ANOVA p≤0.012; Figure 1A, B, and C). There was no difference in AL abundance between the riparian and benthic zones of the Chicago River. Across all three ecosystem types, the dominant types of AL were plastic, paper, and glass (Figure 1D, E, F). Plastic was more abundant in the riparian zone than the beach (ANOVA p = 0.048), but there was no difference in the abundance of paper among locations (ANOVA p = 0.684; Figure 1D, E). Glass was more abundant in the river benthos than the riparian or beach areas (ANOVA p = 0.001; Figure 1F).

### Anthropogenic litter abundance across terrestrial and aquatic ecosystems

**Figure 2:**
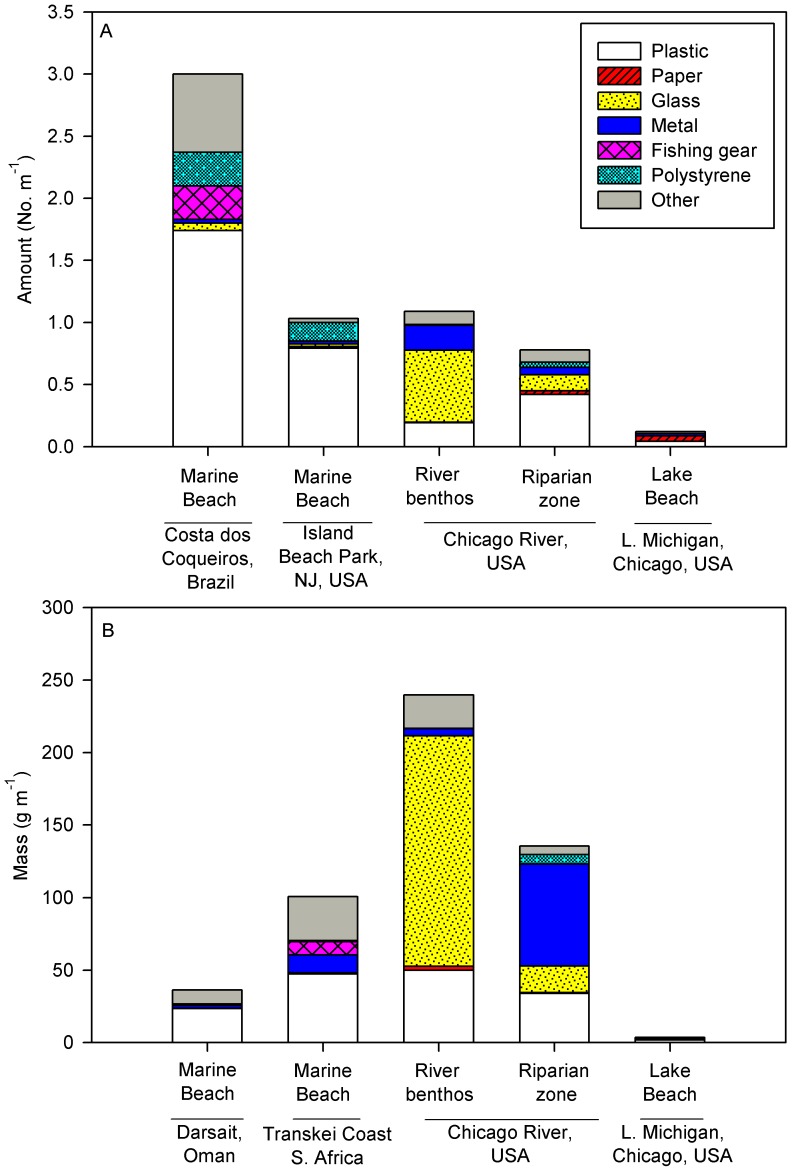
Density (A) and mass (B) of anthropogenic litter (AL) in 2 marine beaches and in 3 habitats from this study: benthic and riparian zones of the North Branch of the Chicago River and a Lake Michigan beach. Data for marine beaches in (A) are from [24] and [23], and data for marine beaches in (B) are from [25] and [26].

The abundance and density of AL at our study sites fall within the range reported in the literature, although values for Lake Michigan beaches were lower than values from two marine sites which used similar methodology and AL categories (Figure 2). While plastic was more abundant in the marine beaches than freshwaters, the mass of plastic was fairly consistent across the 2 marine beaches and the Chicago River riparian and benthic zones (range = 25–55 g m^−1^; Figure 2). The high density of glass in the Chicago River benthos was unique among habitats. Metal represented >50% of the AL mass in the riparian zone, more than other habitats.

**Figure 3:**
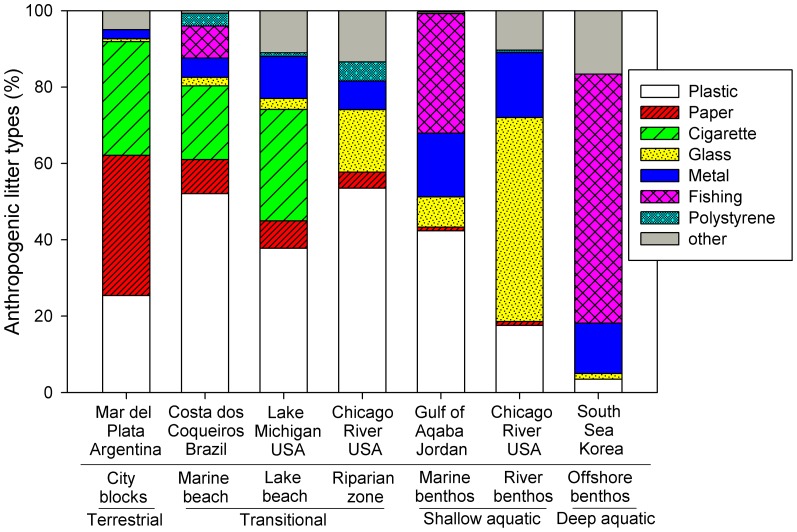
The relative proportion of anthropogenic litter (AL) from our study in the Chicago area (in bold; developed lake beach, riparian zone, and river benthos) relative to city blocks in Argentina [27], a developed marine beach in Brazil [23], a shallow marine benthos site in the Red Sea [1] and an offshore marine benthos site in the South Sea of Korea [29].

Several clear patterns emerged when comparing relative AL abundance across terrestrial, freshwater, and marine sites. Plastic AL represented 17.6–53.5% of total AL in the terrestrial, transitional, and shallow aquatic environments, but was only 3.5% in the offshore benthic environment (50–200 m depth; Figure 3). Paper and cigarette litter was more abundant in terrestrial ecosystems, representing 65% of AL in city blocks, 28–36% of AL on lake and marine beaches, and <1% of AL in the aquatic habitats. AL consisting of fishing items was restricted to marine settings, and increased in abundance from the beach (8.4%) to shallow marine benthos (31.3%) to the offshore marine benthos (65.6%). Metal was generally found in greater abundance in aquatic environments relative to terrestrial or transitional habitats. Finally, glass represented 53.5% of AL in the river benthos, more than other sites.

### Biofilm activity on AL incubated in freshwater ecosystems

**Figure 4:**
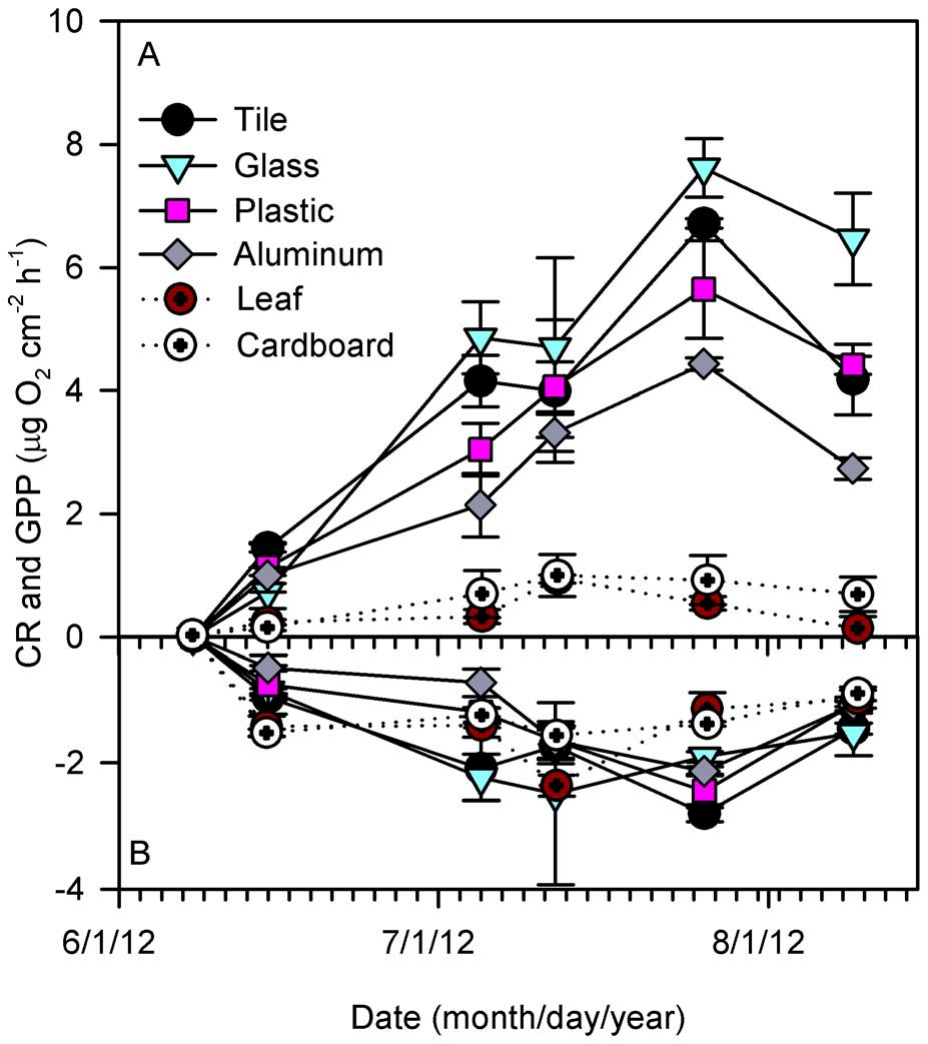
Mean (±SE) gross primary production and community respiration for biofilms colonizing natural substrates (unglazed ceramic tile and leaves) and anthropogenic litter substrates including glass, hard plastic, aluminum, and cardboard, incubated in artificial streams during summer 2012 (N = 18 date^−1^).

AL incubated in artificial streams showed significant variation in GPP through time (RM-ANOVA p<0.001) and among substrates (RM-ANOVA p<0.001; Figure 4A). GPP on cardboard and leaves were not different from each other, but rates were lower than GPP on the hard substrates (glass, aluminum, tile and plastic). On the hard substrates, GPP was highest on glass, aluminum was lowest, and tile and plastic were intermediate. CR was uniform among biofilms for all AL surfaces (RM ANOVA p = 0.075) but rates were different among sampling dates (RM ANOVA p<0.001), attributed to very low rates on the first date (Figure 4B).

**Figure 5:**
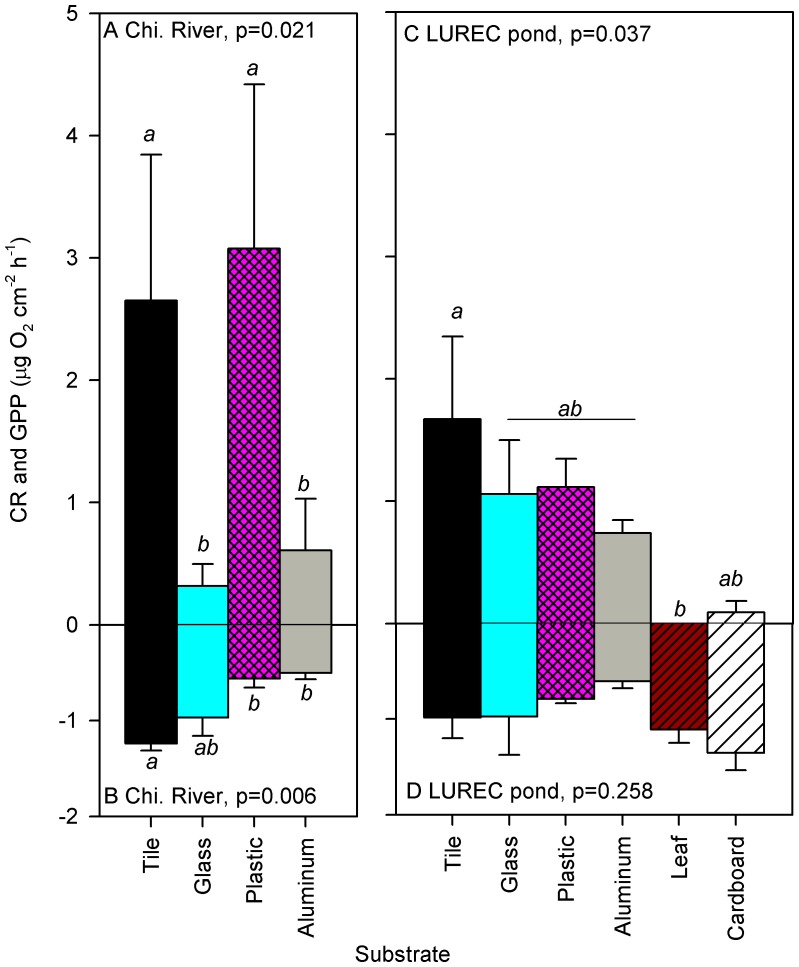
Mean (±SE) gross primary production (GPP) and community respiration (CR) for biofilms colonizing natural substrates (unglazed ceramic tile and leaves) and anthropogenic litter substrates including glass, hard plastic, aluminum, and cardboard incubated in (A) North Branch of the Chicago River, and (B) a pond at Loyola University Retreat and Ecology Campus (LUREC; N = 3 for each substrate at each site). Results (p-values) from 1-way ANOVA among substrates for each metric are shown in each panel, small letters indicate significant differences among substrates (p<0.05) as indicated by Tukey's multiple comparison test.

Biofilm activity on AL surfaces incubated *in situ* showed different patterns in the pond and river sites (Figure 5). GPP on aluminum and glass was lower than tile and plastic in the Chicago River (ANOVA p = 0.021; Figure 5A). Similarly, river biofilms had higher CR on tile than aluminum and plastic, while glass was intermediate (ANOVA p = 0.006; Figure 5B). Organic substrates were completely decomposed, ingested, or fragmented during the incubation in the river. In the pond, biofilm GPP was highest on tile, intermediate on glass, plastic, and aluminum, and lowest on cardboard and leaves (ANOVA p = 0.037, Figure 5C). There was no difference in biofilm CR among substrates in the pond (ANOVA p = 0.258; Figure 5D).

### Bacterial community composition on AL incubated in freshwater ecosystems

**Figure 6:**
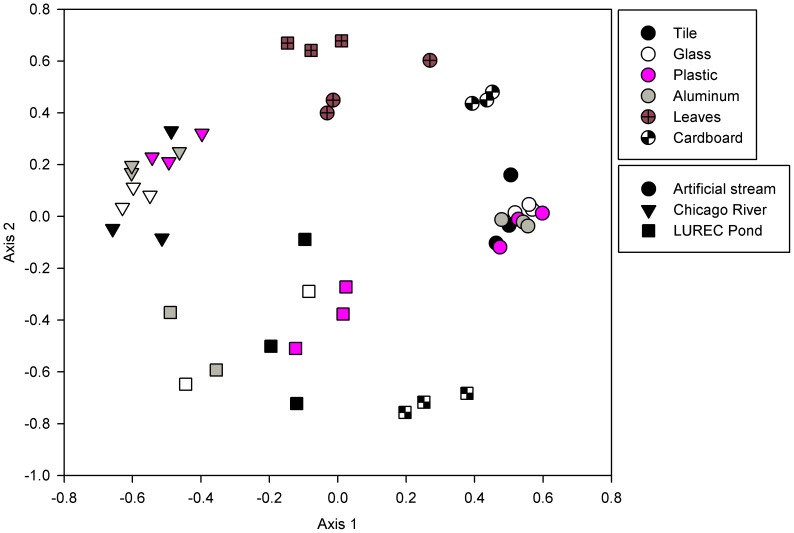
Non-metric multidimensional scaling (nMDS) ordination of bacterial communities based on high-throughput sequencing of bacterial 16S rRNA genes from biofilms colonizing anthropogenic litter (AL) substrates (glass, plastic, aluminum, and cardboard) and natural surfaces (leaves and tile) in artificial streams at Loyola University Chicago, the North Branch of the Chicago River, and a pond at Loyola University Retreat and Ecology Campus (LUREC).

Analysis of biofilm bacterial communities based on high-throughput sequencing of partial 16S rRNA genes demonstrated that all AL substrates were colonized by biofilms containing diverse bacterial assemblages. On average, 1,552 bacterial OTUs were identified per sample. Bacterial community composition was significantly different among the 3 incubation sites. For example, there was a significant effect of site on the number of bacterial OTUs detected within the biofilms (ANOVA, p<0.001), with fewer OTUs detected on average in the artificial stream biofilms (958 OTUs) than in the pond (1,870 OTUs) or river (2,020 OTUs) biofilms. Bacterial communities colonizing substrates incubated in the river, pond and artificial stream were well differentiated on the nMDS ordination (with the exception of the leaf samples from the pond; Figure 6) and communities from the three habitats were significantly different from each other based on ANOSIM (Table S1).

**Figure 7:**
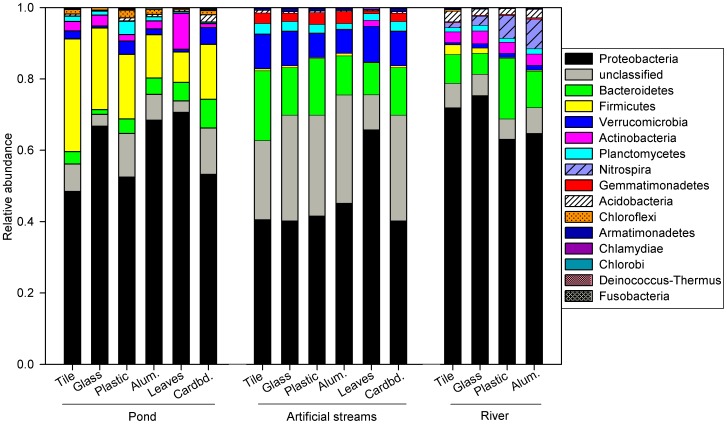
Relative composition of bacteria phyla based on high-throughput sequencing of bacterial 16S rRNA genes from biofilms colonizing anthropogenic litter (AL) substrates (glass, plastic, aluminum, and cardboard) and natural surfaces (leaves and tile) in artificial streams at Loyola University Chicago, the North Branch of the Chicago River, and a pond at Loyola University Retreat and Ecology Campus (LUREC).

The dominant bacterial phyla within the biofilms included Proteobacteria, Bacteroidetes, Firmicutes and Verrucomicrobia, but there were broad differences in the composition of biofilm bacterial communities among sites (Figure 7). For example, river biofilms had significantly higher relative abundances of Acidobacteria and Nitrospira and significantly lower relative abundances of Proteobacteria than the other sites, while the pond biofilms had significantly higher relative abundances of Proteobacteria, Firmicutes and Chloroflexi, and significantly lower relative abundances of Bacteroidetes than the other sites (Table S2). SIMPER analysis identified specific bacterial families that made the most significant contributions to differences in composition of the biofilms across the three sites, and several families were significantly different across sites (Table S3). Examples include significantly higher relative abundance of Burkholderiales, Nitrospiraceae, Nitrosomonadaceae and unclassified Gammaproteobacteria, as well as significantly lower relative abundance of Erythrobacteraceae in the river biofilms as compared to the other sites.

Substrate type also had significant effects on biofilm bacterial community composition. Specifically, biofilm bacterial communities colonizing the organic substrates (cardboard and leaves) in both the pond and artificial stream were clearly distinct from the communities colonizing the hard substrates (glass, plastic, aluminum and tile) on the nMDS ordination (Figure 6, Table S1). We were unable to analyze biofilms colonizing cardboard and leaves in the river because these substrates were no longer present at the conclusion of the incubation. For the pond, river, and artificial stream, the nMDS ordination indicated no differences in bacterial community composition among biofilms on the 4 hard substrates. In addition, there were no significant differences in community composition among the hard substrates in the 3 habitat types (Table S1). Therefore, the biofilm communities colonizing all of the hard substrates were treated as a group by ANOSIM, and were compared to the communities from the biofilms on cardboard and leaves. For both the pond and artificial stream sites, ANOSIM confirmed significant differences in bacterial community composition between the leaf and cardboard substrates and the hard substrates (Table S1). The nMDS (Figure 6) and ANOSIM (Table S1) analyses also indicated that for both the pond and artificial stream sites, the bacterial communities colonizing the cardboard were significantly different from the communities colonizing the leaves (p<0.10).

SIMPER analysis identified the bacterial families making the largest contributions to differences in community composition among the biofilms colonizing the cardboard, leaves and hard substrates (Tables S4 and S5). For the artificial stream, hard substrates had significantly higher relative abundances of Erythrobacteraceae, and the cardboard and leaves had significantly higher relative abundances of Caulobacteraceae (Table S4). Within the artificial stream there were also some significant differences between leaves and cardboard, including significantly higher relative abundances of Cytophagaceae and Opitutaceae on the cardboard, and significantly higher relative abundances of Caulobacteraceae, Rhizobiaceae and Xanthomonadaceae on the leaves (Table S4). For the pond, the biofilm communities colonizing the cardboard and leaves did not show any common differences when compared to the hard substrates; rather each varied from the hard substrates in distinct ways. Specifically, the cardboard had significantly higher relative abundances of Ruminococcaceae and Desulfovibrionaceae than the hard substrates, whereas leaves had higher relative abundances of Oxalobacteraceae, Enterobacteriaceae and Rhizobiaceae than the hard substrates (Table S5). Finally, there was no significant effect of substrate type on community diversity calculated as either Inverse Simpson or Shannon indices (Table S6).

## Discussion

### AL abundance in freshwaters

As expected, we found AL in all freshwater habitats. However, AL density in the river benthos, riparian zone, and lake beaches showed clear differences among ecosystem types, likely driven by differences in hydrology, AL movement and breakdown, and human activity. AL totals on Lake Michigan beaches were unexpectedly low, especially as the beaches studied receive many daily visitors. Low AL density may be attributed to daily beach grooming by the Chicago Park District. Anecdotally, Parks District employees suggested our results for AL density would be much higher if we measured AL collected by the maintenance equipment (C. Breitenbach, personal communication). A more accurate assessment of AL abundance for public beaches will require 1) abatement of AL clean up, or 2) collection of AL from both the beach and from the grooming machines. There are very few studies of AL density on Great Lakes beaches for comparison of our results. High density of small plastic fragments were found on Lake Huron beaches (∼37 pieces m^−2^), however, over 90% were pellets <5 mm in size and required sieving for quantification [39]. Our assessment did not account for AL particles at this scale. More measurements of AL density and composition, across size categories and habitat types, are needed to understand the distribution for AL on Great Lakes beaches.

Total density of AL in the Chicago River's North Branch benthic and riparian zones was similar, but the composition of AL in both habitats suggests that direct littering and variation in the movement and retention of different AL types drive overall abundance. For example, glass was the most abundant AL type found in the river benthos, and consisted mostly of discarded liquor and beer bottles. Our study area was in the Bunker Hill Forest Preserve District of Cook County, located in northwestern Chicago. Like other Forest Preserves, the area is popular for recreation. Glass bottles may have been transported to our study reaches from upstream during floods, but we surmise much of the glass was discarded directly on site. The glass we collected was often partially broken, colonized by microbial biofilms, and contained mud and gravel. Sinking, colonization, and benthic entrainment of glass reduce export from the benthic zone, suggesting that long term accumulation of glass bottles is likely. Local recreation and consumption are also a significant source to AL on some marine beaches [28], but there are few other analyses of AL in rivers to compare our data. A study of proportional AL abundance in a Welsh river found less metal (8%), more plastic (49%), and a large amount of sewage related AL from illegal trash disposal and malfunctioning combined sewer overflows (e.g., feminine hygiene and diapers; 23%) compared to our data [40]. Despite the low number of measurements, riverine sources are reported to contribute up to 80% of global marine AL input [41]–[43]. Our results suggest that selective retention of some AL types in rivers (e.g., glass and metal), combined with the influence of human activity and adjacent land-use, drive benthic AL abundance and could affect estimates for the amount of AL retained and exported in rivers worldwide.

Total AL density in the riparian zone was similar to the river benthos, but the relative composition was different, indicating that riparian AL pools were also determined by mobility and retention. In contrast to the river benthos, there was a trend of more paper and plastic in the riparian zone. Because many of these items were food related, on-site consumption and littering was also probably the major source. One notable pattern was AL accumulation in debris dams and overhanging structures such as bridges, branches, and large woody debris (Figure S1). The orientation of AL suggested that the items were deposited during high flows. This pattern has been called a “Christmas tree” effect elsewhere [40]. Given the propensity for flooding in urban watersheds [44], riparian zone AL is likely to be moved and retained in this fashion elsewhere. A potential AL reduction strategy would be to instruct volunteers to focus on these sites for efficient AL collection. In addition, the repeated wetting and drying of AL at these sites could be an important factor driving its breakdown.

### Relative AL composition across multiple ecosystems

The analysis of relative AL composition of 7 sites spanning different habitats illustrates common trends in AL pools, and allows for inference of critical AL fluxes at each site (Figure 3). One unexpected result of our synthesis was the uniformity in AL composition among city blocks, a marine beach, and the Lake Michigan beach. Cigarette butts contributed a large and relatively even proportion across all 3 sites, and paper was more abundant in these ecosystems than the others in Figure 3. The low abundance of paper and cigarette butts at the aquatic sites suggests that paper and cigarettes 1) have low input rates in aquatic habitats, 2) do not move or accumulate in the same way in aquatic environments as on beaches or city blocks, or 3) rapidly decompose in aquatic habitats. Some combination of all 3 are likely important. For example, after 98 d in the Chicago River, the cardboard was absent from the bags via decomposition, ingestion, or fragmentation, and its incubation in the pond and artificial streams showed a loss of structure after ∼50 days underwater (i.e. cardboard ‘mush’). Cigarette butts consist of paper coverings over cellulose acetate filters, and some brands of have plasticizers added [45]. In terrestrial environments, cigarette butts have a decomposition rate of 0.265 y^−1^ or 3.7 y total (S. Haynes, unpublished data), and can have antimicrobial properties which inhibit biological degradation [45]. To our knowledge, decomposition rates for cigarette butts in aquatic habitats have not been published.

A major factor driving research on AL in the ocean (i.e. marine debris) is the amount of fishing-related garbage found on beaches, shallow marine environments, and the open ocean. Our comparison of AL among 7 sites in Figure 3 supports this pattern, as fishing-related items were found in all 3 marine locations. No fishing-related AL was documented in our freshwater sites and we witnessed no anglers during AL collection. We acknowledge our sampling area encompassed a relatively narrow geographic range. Other freshwater environments may have fishing-related AL, but none have yet been reported in the few published AL studies from rivers and the Great Lakes [40], [42]. The Alliance for the Great Lakes Adopt-a-Beach program has detailed records on AL in Great Lakes beaches since 2003, including measurements of fishing line, nets, and lures. These items typically represent a small proportion of AL on Great Lakes coastal sites, as AL is dominated by food and smoking-related items (J. Cross, personal communication).

The relative proportion of AL across the 7 sites in Figure 3 suggest that AL movement, and not just littering rate, is a critical driver of AL pools in different ecosystems. For example, all 3 benthic sites (river, shallow marine, and offshore marine) and the riparian zone had AL which was more likely to be heavy, including metal, glass, and fishing-related material. This material frequently sinks and may become entrained in the benthos and flooded riparian zones. The river benthos and offshore marine benthos were especially dominated by heavy items. In the river, glass bottles were >50% of the AL items. In the offshore benthos, the largest category was fishing gear (65.2%), which in this case included a combination of heavy materials such as fish pots, nets, octopus jars, and fishing lines [29]. Heavy items the benthic zone of the open ocean originate from direct dumping via fishing activity and not export of terrestrial or riverine sources [29].

Unlike paper items which are degraded more readily, glass and metal are long-lived [20], [46], and likely to be used as habitat. For example, the glass debris we removed from the Chicago River was well-colonized with microbial biofilms, snails, and amphipods (*Gammarus* sp.). Because urban streams can have reduced benthic habitat complexity [44], relatively stable AL items such as glass or tires may represent an important habitat for stream microorganisms and macroinvertebrates. However, AL effects on macroinvertebrate communities are unknown.

A notable trend that emerges from comparison of AL among different studies is that the units used to characterize AL density have a strong impact on the patterns gleaned from the data. For example, glass and metal in the river benthos and riparian zone was abundant and heavy, so the mass of AL at those sites was higher than in the marine or lake beaches, even though the total number of AL items was intermediate (Figure 2). AL has been quantified in various units including abundance (No. m^−1^ or m^−2^), mass (g m^−1^ or m^−2^), and less frequently as surface area (AL cm^2^ habitat m^−2^) [1]. Other ecological metrics are reported in similar units, such as organic matter standing stock (g ash-free dry mass m^−2^), organism density (No. m^−2^), and abundance of large woody debris (m^2^ or m^3^ of wood per habitat area). For AL measurements, the choice of which units are most ecological meaningful may vary depending upon the material. An organic item such as paper may be more usefully expressed in units of mass, as it will likely decompose in similar fashion to leaf litter and represents a potential food resource. In contrast, a hard surface like glass may be more meaningfully expressed in terms of surface area, as the space available for colonization may be the most biologically meaningful aspect of its presence. Finally, the proportion of AL categories present and their relative abundance can be combined into metrics of AL diversity. Given high variation in the types, size, and abundance of AL among habitats, these metrics could synthesize AL “communities” in analogous ways to biological communities [27]. Uniting values into single numbers could also facilitate comparisons better than existing measurements of density or relative proportion.

### Biofilm activity and community composition

When AL substrates were incubated in the three freshwater habitats, all AL became colonized by taxonomically diverse and metabolically active microbial biofilms. Biofilms were dominated by bacteria from the phyla Proteobacteria, Bacteroidetes and Firmicutes, all of which have been identified as common components of freshwater biofilms [47]–[49]. Metabolism rates ranged from −4 µg O_2_ cm^−2^ h^−1^ (CR) to 8 µg O_2_ cm^−2^ h^−1^ (GPP), which aligns with the range reported for the substrate-specific measurements in literature [15], [16].

Site identity significantly affected the activity and composition of microbial biofilms, likely driven by differences in the physical and chemical conditions among sites. For example, biofilms in the artificial stream had higher respiration rates and lower bacterial species (OTU) richness compared to the 2 natural habitats, and mean CR on substrates in the artificial stream was higher than the river and pond. These differences were likely driven by the highly stable conditions in the artificial streams, including higher illumination (due to a lack of shading), higher temperature, and higher rates of nutrient and gas exchange (due to shallow water column and high flow rate) as compared to the field sites. These conditions may have selected for the much higher relative abundance of aerobic, heterotrophic Verrucomicrobia within the artificial streams [50]. In contrast, the higher abundance of bacteria involved in nitrification (e.g., Nitrosomonadaceae and Nitrospira) in the river biofilms was likely the result of higher water column nitrogen concentrations within the Chicago River compared to the pond and artificial stream (T. Hoellein, unpublished data). The most notable feature of the pond biofilm communities was the high relative abundance of Firmicutes, specifically the Planococcaceae, which are a family of aerobic organotrophic bacteria that are known to be halotolerant [51].

AL type affected the activity and composition of microbial biofilms, with the most significant differences observed between organic (leaves and cardboard) and hard substrates (glass, plastic, aluminum and tile). In both the pond and artificial stream, GPP was significantly lower on the organic substrates, suggesting that these communities were dominated by heterotrophic organisms. However, we note the organic substrates were situated inside the mesh bags and shading may have inhibited primary producers. There were also significant differences in bacterial community composition on organic and hard substrates which indicate use of the organic substrates for heterotrophic metabolism. Leaves had high relative abundance of several common plant-associated microbes, including Xanthomonadaceae [52], Rhizobiaceae [53] and Oxalobacteraceae [54]. Leaves in the artificial streams also had high relative abundance of Chitinophagaceae, some species of which have been shown to degrade cellulose [55]. Cardboard also showed very high abundances of bacterial groups with the ability to degrade cellulosic compounds, including Opitutaceae [56] in the artificial stream and Ruminococcaceae [57] in the pond. Cardboard in the pond had high relative abundances of two families of anaerobic bacteria, Desulfovibrionaceae and Ruminococcaceae, suggesting that the low flow conditions may have led to relatively more anaerobic niches than within the shallow, well-mixed artificial streams.

Unexpectedly, there were no significant differences in microbial community composition among tile, glass, plastic, and aluminum within each of the 3 habitats studied. The pattern suggests hard AL surfaces do not appear to affect bacterial community composition. However, patterns of GPP on hard substrates suggest variation in their algal constituents. For example, GPP on aluminum was lower than tiles in the river and artificial streams. The same pattern was observed in the pond (although not statistically significant). Reduced GPP on aluminum relative to the tiles could be attributed to microscopic differences in surface structure or chemistry which could affect algal growth (e.g., fewer attachment points or oxidation). In addition, differences in the patterns of GPP among glass, aluminum, plastic, and tile could be attributable to variation the algal community composition among the 3 ecosystems. Upon visual inspection, biofilms on all inorganic AL items in the river and pond had a brown and green appearance, with little evidence of strands of filamentous algae. In contrast, most substrates in the artificial stream had visible filamentous algae. We did not quantify AL surface texture, algal community composition, or chlorophyll *a*. However, our results suggest those analyses could be an important component of future studies which analyze the effect of AL on freshwater biofilms.

Differences in microbial community composition among habitats and AL types suggest AL movement could affect dispersal of microbial biofilm constituents among connected aquatic ecosystems. For example, biofilms colonizing AL surfaces such as plastic, which is mobile and slow degrading, may be more likely to be transported downstream intact than those on natural surfaces such as rocks (largely stationary) and leaf litter or fine sediment (which decompose). River biofilms had significantly higher Nitrosomonadaceae and Nitrospira bacterial taxa which carry out nitrification. Downstream transport of riverine AL over long distances to lake or marine ecosystems could deliver intact microbial communities through this novel mechanism, thereby affecting community structure and function in the receiving water body. This process has been demonstrated for dispersal of invasive species in the marine environment [58], but not for movement of organisms which colonize AL in freshwaters.

### Revised conceptual model for AL ecology with a cross-ecosystem perspective

**Figure 8:**
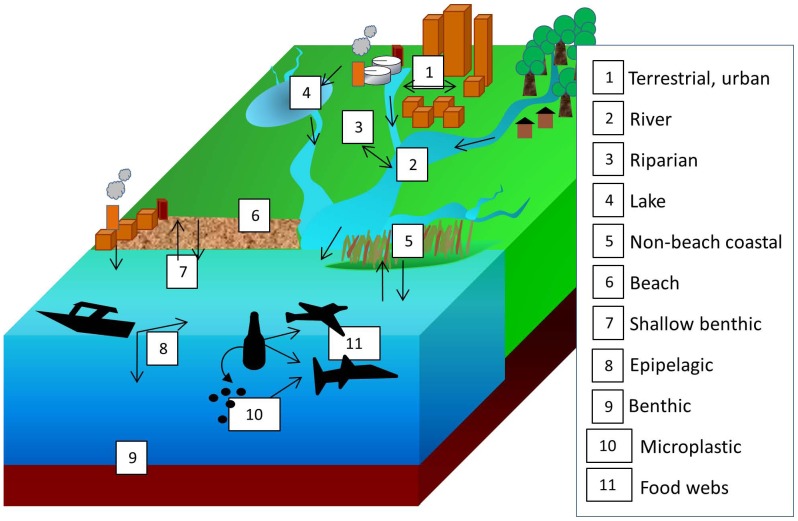
Conceptual diagram of major pools and fluxes of anthropogenic litter (AL) in terrestrial, freshwater, and marine ecosystems, modified from [10]. This model unites the early stages of the AL “life cycle,” in terrestrial and freshwater ecosystems to stages of accumulation and transformation in coastal and pelagic environments.

While AL in the marine environment is well documented [1], [4], [46], research on AL abundance and its role(s) in freshwater and terrestrial ecosystems lags far behind. We suggest this discrepancy may be attributed in part to an issue of nomenclature. The term “marine debris” has been used to describe AL, even when it is found in non-marine ecosystems [59]. Other studies have used disparate terms such as urban litter, riverine litter, floatables, and solid waste to refer to terrestrial or freshwater AL [22], [27], [40], [42]. We suggest marine-focused terminology for ocean research, and lack of unity in terminology for research in other ecosystems may inhibit a more complete understanding of the sources and fate of AL at landscape and global scales. We suggest the term anthropogenic litter is most useful because 1) AL differentiates the material from natural litter or debris accumulations (e.g. leaf litter or woody debris), 2) AL describes the material independent of its collection site, and 3) the term could promote an expanded perspective on the spatial dynamics and entire ‘life cycle’ of AL to unify terrestrial, freshwater, and marine ecosystem research on the topic (Figure 8).

We composed a conceptual model to guide our AL research by building on the diagram for the study of marine plastics, which shows 3 pools for marine plastic including beaches, coastal waters/sediments, and the open ocean [10]. We expanded this perspective by modifying the vocabulary (i.e. AL rather than marine debris) and adding several pools: 1) terrestrial environments, 2) rivers, 3) riparian zones, 4) lakes, and 5) marshes or other non-beach coastal habitats (Figure 8). Within the marine environment, we can separate major AL pools into 7) shallow benthos, 8) epipelagic, 9) benthic, 10) microplastic, and 11) food webs (i.e. consumption by microbes and animals).

Expanding the number of pools in the AL conceptual diagram also requires the consideration of fluxes not conventionally incorporated into models of AL movement [10]. Fluxes of AL are mediated by abiotic factors such as wind, flooding, currents, retention, and buoyancy. In addition, biotic activity (e.g., colonization, decomposition, and ingestion), and human activity affect AL flux [60]. The relative importance of these fluxes is likely to differ among terrestrial, freshwater, and marine ecosystems. For example, our data for AL pools in freshwater ecosystems suggest human activity can drive both AL input (i.e. littering), and output (i.e. beach maintenance). Some pools in Figure 8 are likely to be net sinks of AL, or sites of AL accumulation over very long time scales. Other pools are likely to represent locations where AL is only temporarily stored before it is moved, broken down, or consumed. Differentiating the net sinks from temporary storage sites requires research which incorporates measurements of biotic, abiotic, and anthropogenic fluxes along with AL pools. Building a greater understanding of the entire AL “life cycle” remains a major challenge for the science of AL ecology, but will serve as a critical tool for reducing its continued recruitment and mitigating its environmental impacts.

## Supporting Information

Figure S1 Anthropogenic litter (AL) deposited on vegetation overhanging the North Branch of the Chicago River.
(TIF)

Table S1 ANOSIM analysis of differences in bacterial community composition by sites and across substrate types. ‘Hard substrates’ includes tile, glass, plastic, and aluminum.
(DOCX)

Table S2 Relative abundances of most abundant bacterial phyla across sampling sites.
(DOCX)

Table S3 Relative abundances of bacterial families making the largest contributions to variations in biofilm composition across sampling sites.
(DOCX)

Table S4 Relative abundances of bacterial families within artificial stream biofilms making the largest contribution to variations between substrate types.
(DOCX)

Table S5 Relative abundances of bacterial families within pond biofilms making the largest contribution to variations between substrate types.
(DOCX)

Table S6 Mean (±SE) values for the Inverse Simpson and Shannon diversity (H′) indices for microbial communities colonizing substrates in the three study sites.
(DOCX)
