# Supplementary figures and images for: Anthropogenic Litter in Urban Freshwater Ecosystems: Distribution and Microbial Interactions
Source: PLoS One. 2014 Jun 23;9(6):e98485. doi: 10.1371/journal.pone.0098485 (PMC4067278; doi:10.1371/journal.pone.0098485)

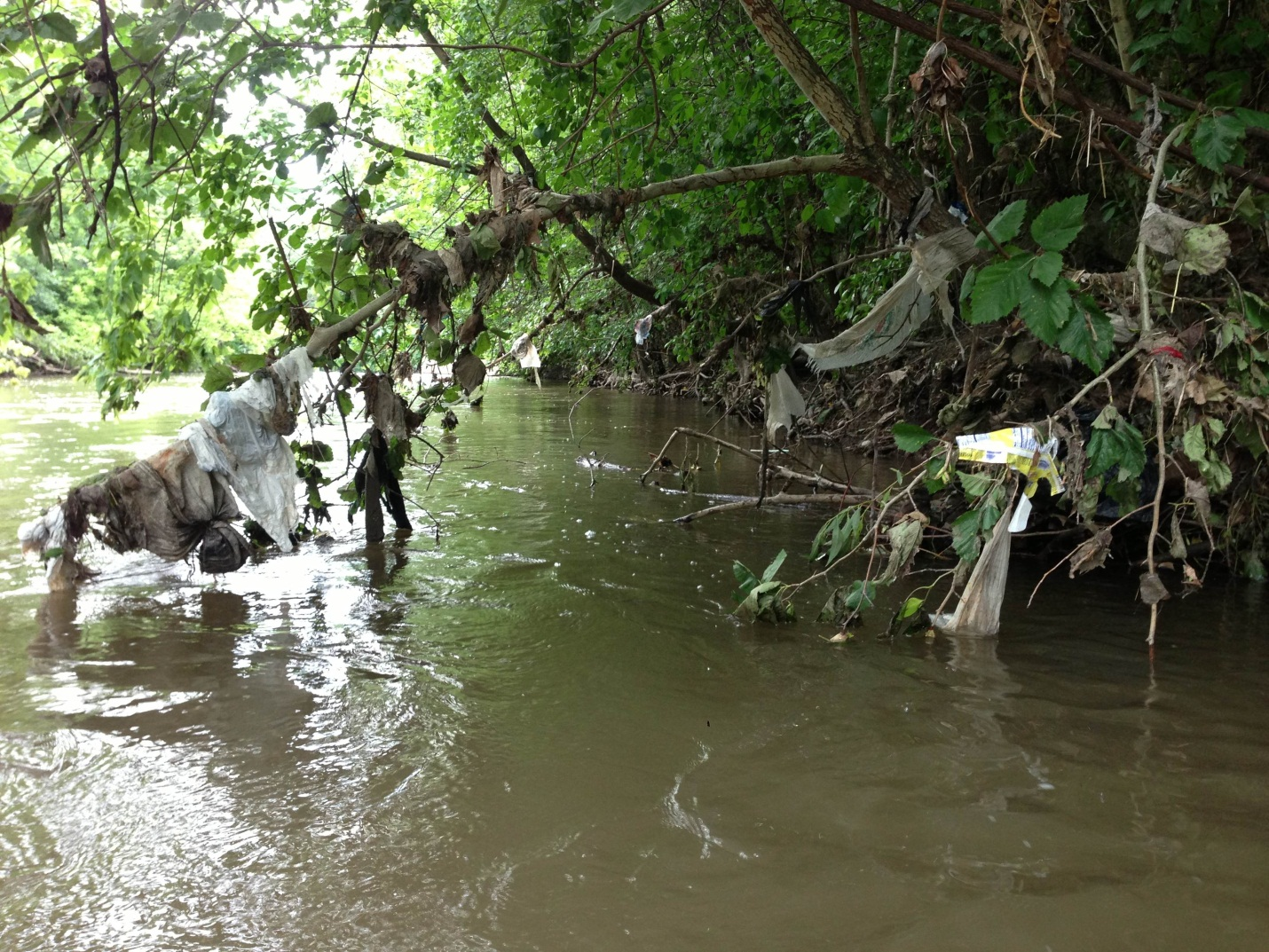

Supplement: Figure S1 — Anthropogenic litter (AL) deposited on vegetation overhanging the North Branch of the Chicago River. (TIF) [file pone.0098485.s001.tif]
