# Supplementary material for: Anthropogenic Litter in Urban Freshwater Ecosystems: Distribution and Microbial Interactions
Source: PLoS One. 2014 Jun 23;9(6):e98485. doi: 10.1371/journal.pone.0098485 (PMC4067278; doi:10.1371/journal.pone.0098485)
Supplement: Table S1 — ANOSIM analysis of differences in bacterial community composition by sites and across substrate types. ‘Hard substrates’ includes tile, glass, plastic, and aluminum. (DOCX) [file pone.0098485.s002.docx]

| **Comparison** | **R** | **P** |
| --- | --- | --- |
| *All substrates* |  |  |
| Pond, river | 0.545 | <0.001 |
| Pond, artificial stream | 0.661 | <0.001 |
| River, artificial stream | 0.986 | <0.001 |
| *Organic vs. hard substrates* |  |  |
| Pond: cardboard, hard substrates | 0.954 | 0.004 |
| Pond: leaves, hard substrates | 0.518 | 0.014 |
| Pond: cardboard, leaves | 1.000 | 0.088 |
| Artificial stream: cardboard, hard substrates | 1.000 | 0.004 |
| Artificial stream: leaves, hard substrates | 0.997 | <0.001 |
| Artificial stream: cardboard, leaves | 0.926 | 0.095 |
| *Chicago River hard substrates* |  |  |
| Tile, glass | 0.037 | 0.501 |
| Tile, plastic | 0.185 | 0.298 |
| Tile, aluminum | 0.148 | 0.211 |
| Glass, plastic | 0.852 | 0.106 |
| Glass, aluminum | 0.556 | 0.101 |
| Plastic, aluminum | 0.111 | 0.390 |
| *Artificial stream hard substrates* |  |  |
| Tile, glass | 0.444 | 0.092 |
| Tile, plastic | -0.074 | 0.607 |
| Tile, aluminum | 0.370 | 0.091 |
| Glass, plastic | -0.037 | 0.598 |
| Glass, aluminum | 0.630 | 0.097 |
| Plastic, aluminum | 0.037 | 0.496 |
| *LUREC pond hard substrates* |  |  |
| Tile, glass | -0.167 | 0.793 |
| Tile, plastic | 0.000 | 0.489 |
| Tile, aluminum | -0.083 | 0.606 |
| Glass, plastic | 0.167 | 0.208 |
| Glass, aluminum | -0.500 | 1.000 |
| Plastic, aluminum | 0.500 | 0.100 |
