# Supplementary material for: Anthropogenic Litter in Urban Freshwater Ecosystems: Distribution and Microbial Interactions
Source: PLoS One. 2014 Jun 23;9(6):e98485. doi: 10.1371/journal.pone.0098485 (PMC4067278; doi:10.1371/journal.pone.0098485)
Supplement: Table S2 — Relative abundances of most abundant bacterial phyla across sampling sites. (DOCX) [file pone.0098485.s003.docx]

| **Phyla** | **Artificial Stream (%)** | **Chicago River (%)** | **LUREC Pond (%)** | **p value^†^** |
| --- | --- | --- | --- | --- |
| Acidobacteria | 0.39^a‡^ | 2.14^b^ | 0.86^a^ | <0.001 |
| Actinobacteria | 0.54^a^ | 3.21^b^ | 3.63^b^ | <0.001 |
| Bacteroidetes | 14.15^a^ | 10.28^a^ | 4.62^b^ | <0.001 |
| Chloroflexi | 0.06^a^ | 0.17^a^ | 1.19^b^ | <0.001 |
| Firmicutes | 0.42^a^ | 1.19^a^ | 18.19^b^ | <0.001 |
| Gemmatimonadetes | 2.38^a^ | 0.32^b^ | 0.03^b^ | <0.001 |
| Nitrospira | 0.00^a^ | 4.67^b^ | 0.02^a^ | <0.001 |
| Proteobacteria | 45.85^c^ | 68.84^a^ | 59.04^b^ | <0.001 |
| Verrucomicrobia | 11.23^a^ | 1.09^b^ | 2.40^b^ | <0.001 |

^†^p value for site effect based on ANOVA

^‡^data points followed by different letters are significantly different (p<0.05) among sites based on Tukey's post-hoc test.
