# Supplementary material for: Anthropogenic Litter in Urban Freshwater Ecosystems: Distribution and Microbial Interactions
Source: PLoS One. 2014 Jun 23;9(6):e98485. doi: 10.1371/journal.pone.0098485 (PMC4067278; doi:10.1371/journal.pone.0098485)
Supplement: Table S3 — Relative abundances of bacterial families making the largest contributions to variations in biofilm composition across sampling sites. (DOCX) [file pone.0098485.s004.docx]

| **Taxon** | **Artificial Stream (%)** | **Chicago River (%)** | **LUREC Pond (%)** | **p value^†^** |
| --- | --- | --- | --- | --- |
| Burkholderiales family | 0.61^a‡^ | 5.71^b^ | 1.00^a^ | <0.001 |
| Erythrobacteraceae | 4.70^a^ | 0.12^b^ | 3.76^a^ | 0.002 |
| Nitrosomonadaceae | 0.00^a^ | 0.11^b^ | 0.00^a^ | <0.001 |
| Nitrospiraceae | 0.00^a^ | 4.67^b^ | 0.02^a^ | <0.001 |
| Planococcaceae | 0.02^a^ | 0.08^a^ | 9.59^b^ | <0.001 |
| Spartobacteria family | 6.95^a^ | 0.08^b^ | 0.38^b^ | <0.001 |
| unclassified Bacteria | 22.03^a^ | 6.42^b^ | 8.10^b^ | <0.001 |
| unclassified Betaproteobacteria | 0.56^a^ | 0.02^b^ | 1.74^c^ | <0.001 |
| unclassified Gammaproteobacteria | 0.36^a^ | 8.00^b^ | 1.22^a^ | <0.001 |

^†^p value for site effect based on ANOVA

^‡^data points followed by different letters are significantly different (p<0.05) among sites based on Tukey's post-hoc test.
