# Supplementary material for: Anthropogenic Litter in Urban Freshwater Ecosystems: Distribution and Microbial Interactions
Source: PLoS One. 2014 Jun 23;9(6):e98485. doi: 10.1371/journal.pone.0098485 (PMC4067278; doi:10.1371/journal.pone.0098485)
Supplement: Table S4 — Relative abundances of bacterial families within artificial stream biofilms making the largest contribution to variations between substrate types. (DOCX) [file pone.0098485.s005.docx]

| **Family** | **Cardboard (%)** | **Leaves (%)** | **Hard Substrates (%)** | | **p value^†^** | |
| --- | --- | --- | --- | --- | --- | --- |
| Caulobacteraceae | 1.32^a‡^ | 5.70^b^ | 0.17^c^ | <0.001 | |  |
| Chitinophagaceae | 3.28^a^ | 5.71^b^ | 2.30^a^ | 0.001 | |  |
| Cytophagaceae | 5.38^a^ | 0.06^b^ | 4.54^a^ | 0.022 | |  |
| Erythrobacteraceae | 0.90^a^ | 0.85^a^ | 6.61^b^ | <0.001 | |  |
| Opitutaceae | 14.88^a^ | 0.16^b^ | 0.08^b^ | <0.001 | |  |
| Rhizobiaceae | 1.80^a^ | 14.83^b^ | 0.08^a^ | <0.001 | |  |
| Spartobacteria family | 9.04 | 8.37 | 6.06 | 0.476 | |  |
| Xanthomonadaceae | 0.46^a^ | 3.35^b^ | 0.92^a^ | <0.001 | |  |

^†^p value for effect of substrate type based on ANOVA

^‡^data points followed by different letters are significantly different (p<0.05) among substrate types based on Tukey's post-hoc test.
