# Supplementary material for: Anthropogenic Litter in Urban Freshwater Ecosystems: Distribution and Microbial Interactions
Source: PLoS One. 2014 Jun 23;9(6):e98485. doi: 10.1371/journal.pone.0098485 (PMC4067278; doi:10.1371/journal.pone.0098485)
Supplement: Table S5 — Relative abundances of bacterial families within pond biofilms making the largest contribution to variations between substrate types. (DOCX) [file pone.0098485.s006.docx]

| **Family** | **Cardboard (%)** | **Leaves (%)** | **Hard Substrates (%)** | **p value^†^** |
| --- | --- | --- | --- | --- |
| Desulfovibrionaceae | 7.86^a‡^ | 0.12^b^ | 0.08^b^ | <0.001 |
| Enterobacteriaceae | 0.01^a^ | 6.58^b^ | 0.03^a^ | 0.004 |
| Erythrobacteraceae | 0.17 | 2.53 | 5.21 | 0.230 |
| Oxalobacteraceae | 0.03^a^ | 6.84^b^ | 0.06^a^ | 0.000 |
| Planococcaceae | 0.15 | 4.64 | 13.91 | 0.144 |
| Rhizobiaceae | 0.18^a^ | 6.34^b^ | 0.17^a^ | 0.001 |
| Rhodobacteraceae | 1.29 | 3.60 | 9.55 | 0.271 |
| Ruminococcaceae | 11.84^a^ | 0.05^b^ | 1.02^b^ | 0.001 |
| Sphingomonadaceae | 0.47 | 10.48 | 6.05 | 0.158 |

^†^p value for effect of substrate type based on ANOVA

^‡^data points followed by different letters are significantly different (p<0.05) among substrate types based on Tukey's post-hoc test.
