# Supplementary material for: Anthropogenic Litter in Urban Freshwater Ecosystems: Distribution and Microbial Interactions
Source: PLoS One. 2014 Jun 23;9(6):e98485. doi: 10.1371/journal.pone.0098485 (PMC4067278; doi:10.1371/journal.pone.0098485)
Supplement: Table S6 — Mean (±SE) values for the Inverse Simpson and Shannon diversity (H′) indices for microbial communities colonizing substrates in the three study sites. (DOCX) [file pone.0098485.s007.docx]

|  | **Inverse Simpson** | | |  | **H'** | | |
| --- | --- | --- | --- | --- | --- | --- | --- |
| **Substrate** | **Mean** | **SE** | **p** |  | **Mean** | **SE** | **p** |
| *Chicago River* | |  |  |  |  |  |  |
| Tile | 50.3 | 4.92 | 0.322 |  | 4.97 | 0.21 | 0.246 |
| Glass | 36.5 | 6.14 |  |  | 5.20 | 0.17 |  |
| Plastic | 36.7 | 1.64 |  |  | 5.07 | 0.12 |  |
| Aluminum | 32.7 | 10.46 |  |  | 5.45 | 0.14 |  |
|  |  |  |  |  |  |  |  |
| *LUREC Pond* | |  |  |  |  |  |  |
| Tile | 34.6 | 6.85 | 0.738 |  | 4.24 | 1.11 | 0.066 |
| Glass | 50.1 | 11.44 |  |  | 3.67 | 0.62 |  |
| Plastic | 13.1 | 1.89 |  |  | 5.59 | 0.22 |  |
| Aluminum | 19.1 | 2.34 |  |  | 4.98 | 0.26 |  |
| Leaves | 37.2 | 11.70 |  |  | 5.88 | 0.06 |  |
| Cardboard | 39.1 | 34.36 |  |  | 3.64 | 0.09 |  |
|  |  |  |  |  |  |  |  |
| *Artificial streams* | |  |  |  |  |  |  |
| Tile | 20.4 | 1.51 | 0.353 |  | 4.28 | 0.10 | 0.422 |
| Glass | 23.3 | 5.80 |  |  | 4.02 | 0.07 |  |
| Plastic | 18.0 | 2.04 |  |  | 4.07 | 0.14 |  |
| Aluminum | 15.9 | 3.10 |  |  | 4.10 | 0.15 |  |
| Leaves | 22.1 | 5.24 |  |  | 4.31 | 0.15 |  |
| Cardboard | 28.7 | 4.41 |  |  | 3.99 | 0.15 |  |

^†^p value for effect of substrate type based on ANOVA

^‡^data points followed by different letters are significantly different (p<0.05) among substrate types based on Tukey's post-hoc test.
